# Do plants mediate their anti-diabetic effects through anti-oxidant and anti-apoptotic actions? an *in vitro* assay of 3 Indian medicinal plants

**DOI:** 10.1186/1472-6882-13-257

**Published:** 2013-10-05

**Authors:** Samidha A Kalekar, Renuka P Munshi, Urmila M Thatte

**Affiliations:** 1 Department of Clinical Pharmacology, TN Medical College and BYL Nair Charitable Hospital, Mumbai Central, Mumbai 400 008, India; 2 Department of Clinical Pharmacology, Seth GS Medical College & KEM Hospital, Parel, Mumbai 400 012, India

**Keywords:** Diabetes, Streptozotocin, RINm5F cells, Anti-oxidant, Anti-apoptotic, *Phyllanthus emblica*, *Curcuma longa*, *Tinospora cordifolia*

## Abstract

**Background:**

Both experimental and clinical studies suggest that oxidative stress plays a major role in the pathogenesis of both types of diabetes mellitus. This oxidative stress leads to β-cell destruction by apoptosis. Hence exploring agents modulating oxidative stress is an effective strategy in the treatment of both Type I and Type II diabetes. Plants are a major source of anti-oxidants and exert protective effects against oxidative stress in biological systems. *Phyllanthus emblica, Curcuma longa* and *Tinospora cordifolia* are three such plants widely used in *Ayurveda* for their anti-hyperglycemic activity. Additionally their anti-oxidant properties have been scientifically validated in various experimental *in vitro* and *in vivo* models. Hence the present *in vitro* study was planned to assess whether the anti-hyperglycemic effects of the hydro-alcoholic extracts of *Phyllanthus emblica (Pe)* and *Curcuma longa (Cl)* and aqueous extract of *Tinospora cordifolia (Tc)* are mediated through their antioxidant and/or anti-apoptotic property in a streptozotocin induced stress model.

**Methods:**

RINm5F cell line was used as a model of pancreatic β-cells against stress induced by streptozotocin (2 mM). Non-toxic concentrations of the plant extracts were identified using MTT assay. Lipid peroxidation through MDA release, modulation of apoptosis and insulin release were the variables measured to assess streptozotocin induced damage and protection afforded by the plant extracts.

**Results:**

All 3 plants extracts significantly inhibited MDA release from RIN cells indicating protective effect against STZ induced oxidative damage. They also exhibited a dose dependent anti-apoptotic effect as seen by a decrease in the sub G0 population in response to STZ. None of the plant extracts affected insulin secretion from the cells to a great extent.

**Conclusion:**

The present study thus demonstrated that the protective effect of the selected medicinal plants against oxidative stress induced by STZ *in vitro*, which was exerted through their anti-oxidant and anti-apoptotic actions.

## Background

Diabetes mellitus is a complex metabolic disorder resulting from either insulin insufficiency or insulin dysfunction. Type I diabetes (insulin dependent), is distinguishable by selective destruction, *via* an autoimmune process, of the insulin-secreting β-cell in the pancreatic islets of Langerhans, and pancreatic β-cells are thought to be destroyed by apoptotic death [1]. Type II diabetes or non-insulin-dependent diabetes mellitus (NIDDM), is characterized by insulin resistance, in which the primary insulin target organs (adipose, muscle, and liver tissues) are poorly responsive to insulin action and which may be combined with reduced insulin secretion caused by a progressive loss of β-cell function [2]. Oxidative stress plays a major role in the pathogenesis of both types of diabetes mellitus [3]. Previous pre-clinical and clinical studies have demonstrated that the elevation of reactive oxygen species (ROS) due to oxidative stress is associated with decreased antioxidant capacity in the islet β-cells in type 1 and type 2 diabetic subjects [4,5]. The pancreatic β-cells are susceptible to oxidative stress leading to cell apoptosis and consequent insulin secretion reduction [6,7]. Hence evaluating agents that modulate oxidative stress is an essential step for the future development of therapeutic strategies for both Type I and Type II diabetes.

Antioxidants, both exogenous and endogenous, whether synthetic or natural, can be effective in prevention of oxidative stress and protection of β-cell loss. Plants have been suggested as the major source of anti-oxidants and are capable of exerting protective effects against oxidative stress in biological systems [8]*. Phyllanthus emblica, Curcuma longa* and *Tinospora cordifolia* are three such plants that are widely used in *Ayurveda* for their anti-hyperglycemic activity and their anti-oxidant properties have been scientifically validated in various experimental *in vitro* and *in vivo* models [9-14].

The present study was thus conducted to evaluate the protective effect of the selected medicinal plants against the oxidative stress induced by streptzotocin (STZ) using RINm5F cells. RINm5F cell line is insulin secreting pancreatic beta cell line widely used as an alternative model instead of animals to screen agents for anti-diabetic effects of plants and β-cell dysfunction. STZ acts on β-cells by generation of various ROS and act partially through oxidative stress to induce β-cell apoptosis that leads to the loss of β-cell mass and activation of [poly (ADP-ribose) polymerase (PARP) leading to decrease in insulin secretion [15,16].

The effect of plants on the oxidative stress was evaluated using variables like lipid peroxidation in terms of Malondialdehyde (MDA) release, modulation of apoptosis and insulin release depending on the mechanism of action of STZ (to assess the damage induced by streptozotocin). Glibenclamide, a known anti-diabetic agent was used as a positive control to compare the effect of plants. Through this study an attempt was also made to evaluate whether anti-hyperglycemic activity exhibited by these plants is mediated through their antioxidant and/or anti-apoptotic property. This will facilitate in exploring the mechanistic activity of the selected plants which will open up avenues for development of these plants as anti-diabetic agents.

## Methods

### Materials

All chemicals were purchased from Sigma (St Louis, MO, USA) and all culture media, serum supplements and antibiotic mixture solutions were purchased from Gibco BRL Life Technologies Inc. (Carlsbad, CA, USA) unless otherwise indicated.

### Study drugs

Standardized hydroalcoholic extracts of *Phyllanthus emblica* (fruits) and *Curcuma longa* (roots) and aqueous extract of *Tinospora cordifolia* (stem) in powder form were procured from Natural Remedies, Bangalore. The authentication report and Certificate of analysis is available on file. Pure powder of Glibenclamide was obtained as a gift from Glenmark Pharmaceuticals, Mumbai.

The plant extracts were evaluated over a concentration range from 2.5 to 100 μg/ml. These concentrations were calculated from the therapeutic dose of the plants and were within the concentration range commonly used for *in vitro* studies. Glibenclamide was evaluated at a dose of 1 μg/ml; dose extrapolated from the median therapeutic human dose.

The extracts of *Phyllanthus emblica* and *Tinospora cordifolia* were dissolved in RPMI, cell culture medium and diluted further. *Curcuma longa* and the standard drug was first dissolved in DMSO and then reconstituted in cell culture medium to achieve the required concentration. The concentration of DMSO in the extract and standard drug did not exceed 0.2%, which had no effect on the obtained results.

#### ***Cell culture***

A rat insulinoma cell line (RINm5F) was purchased from National Centre for Cell Sciences, Pune and maintained at 37°C under a humidified, 5% CO2 atmosphere in RPMI-1640 medium supplemented with 10% fetal bovine serum, 100 units/ml of penicillin, and 100 μg/ml of streptomycin, and the medium was changed every two days. Cells from passages 20–60 were used.

#### ***Methodology***

##### 

**Cell viability assay** Viability of RINm5F cells after treatment with the plant extracts and standard drug was determined by assaying for the reduction of 3-(4,5-dimethylthiazol- 2-yl)-2,5-diphenyltetrazolium bromide (MTT) to formazan. Briefly, cells were seeded in 96-well plates (1 × 10^4^ cells per well in 200 μl of medium followed by treatment with different concentrations (2.5, 5,10,25,50 and 100 μg/ml) of the selected plant extracts. The plate was incubated for 24 hrs at 37°C with 5% CO_2._ Following incubation, MTT was added for 4 h followed by the addition of HCl:Isopropanol (1:24) to lyse the cells and solubilize the formazan crystals. The absorbance of the resulting solution was read using an ELISA plate reader (Biorad Laboratories Pvt Ltd., India) at a wavelength of 570 nm. The amount of color produced was directly proportional to the number of viable cells. Concentrations of the extracts that did not affect cell viability were selected for further experiments.

##### 

**Assay procedure** RINm5F cells were grown in RPMI-1640 medium supplemented with 10% FBS, in a humidified atmosphere of 5% CO_2_ at 37°C. On attaining 75-80% confluency, the cells, at a concentration of 1 × 10^6^cells/ml, were loaded in a 24 well plate. The cells were then treated with different concentrations of plant extracts selected from viability study and standard anti-diabetic drug in presence/absence of STZ (2 mM). Untreated cells served as control. The plate was then incubated for 24 hrs at 37°C in presence of 5% CO_2_. At the end of incubation, effect of the plants against STZ induced oxidative stress was assessed on the select variables.

##### 

**Measurement of lipid peroxidation** Lipid peroxidation was measured in terms of levels of malondialdehyde (MDA) expressed as nmole/mg of protein of the cell suspension. After the incubation levels (MDA) were measured by Thiobarbituric acid method [17].RINm5F cells were re suspended in 0.5 ml of ice-cold Phosphate Buffered Saline (PBS) and sonicated for 30 seconds. The assay mixture contained 1 ml of 0.5 M KCl in 10 mM Tris HCl and 0.5 ml of 52 mM Thiobarbituric acid (TBA). The assay mixture was then heated to 80°C for 30 mins and after cooling to 0°C was centrifuged at 800×g for 10 minutes. The absorbance of the supernatant was measured at 532 nm using a dual beam spectrophotometer (Analytika Jena, Germany). The levels of MDA was calculated based on standard curve constructed with 1,13,3- tetra ethoxy propane. Cell protein was estimated using Folin Lowry method and extrapolated from standard curve constructed with Bovine Serum Albumin (BSA).

##### 

**Measurement of apoptosis** The degree of apoptosis was determined using Propidium iodide (PI) stain which intercalates into double stranded nucleic acids that can be excited by 488 nm lasers [18]. The cells, subjected to treatment were lysed using a lysis buffer containing Tris HCl (100 mM), NaCl (154 mM), CaCl_2_ (1 mM), MgCl_2_ (0.5 mM) and 0.1% NP 40. Propidium iodide (50 μg/ml) was added to the lysing solution to stain the cells. RNAse (40 μg/ml) was also added, as PI is known to intercalate with RNA. The lysing solution containing the cells was then incubated in dark at room temperature for 30 minutes and the fluorescent intensity was measured using a FAC-Scan (Becton Dickinson, San Jose, CA).

##### 

**Measurement of insulin secretion** The supernatant of the cells after each incubation was collected and used to measure the insulin secretion using a Rat Insulin Elisa kit (Mercodia, Sweden).

##### 

**Statistical analysis** The results were analyzed for statistical significance by one-way analysis of variance (ANOVA) test followed by Tukey's post hoc test using the Graphpad Instat software (version 3.06). All data was expressed as Mean ± SD values. In all analyses, a p value of <0.05 was considered statistically significant.

## Results & discussion

A number of medicinal plants have been used to control blood glucose and to either inhibit or trigger fundamental cellular processes mainly through their anti-oxidant potential, making oxidative stress and apoptosis acquiescent to pharmacological intervention [19].In our study, we evaluated the protective effect of the selected medicinal plants to modulate the oxidative stress and apoptosis which are the major contributors to diabetes mellitus development. Furthermore, the effect of plants on the modulation of insulin release was also studied.

Prior to evaluating the effect of plants, viability studies were carried out. The viability studies help in eliminating the cytotoxic doses of plant extracts and thereby determining out the precise range of concentrations of the extracts for further experiments. The viability of RINm5F cells after treatment with the plant extracts was assayed by the MTT assay over a concentration range of 2.5 to 100 μg/ml. On evaluating the MTT results, it was observed that*, Phyllanthus emblica* and *Tinospora cordifolia* did not affect the viability of RIN cells up to 100 μg/ml, whereas *Curcuma longa* affected viability at higher concentration. Hence efficacy studies were carried out using a concentration range of 2.5 to 100 μg/ml for *Phyllanthus emblica* and *Tinospora cordifolia* and 50 μg/ml for Curcuma *longa*.

Following viability study, the effect of plant extracts was evaluated on the select variables based on therapeutic interest to assess the anti-oxidant, anti-apoptotic and insulin secretagogue effect of the plant extracts.

### Effect on lipid peroxidation

**Figure 1:**
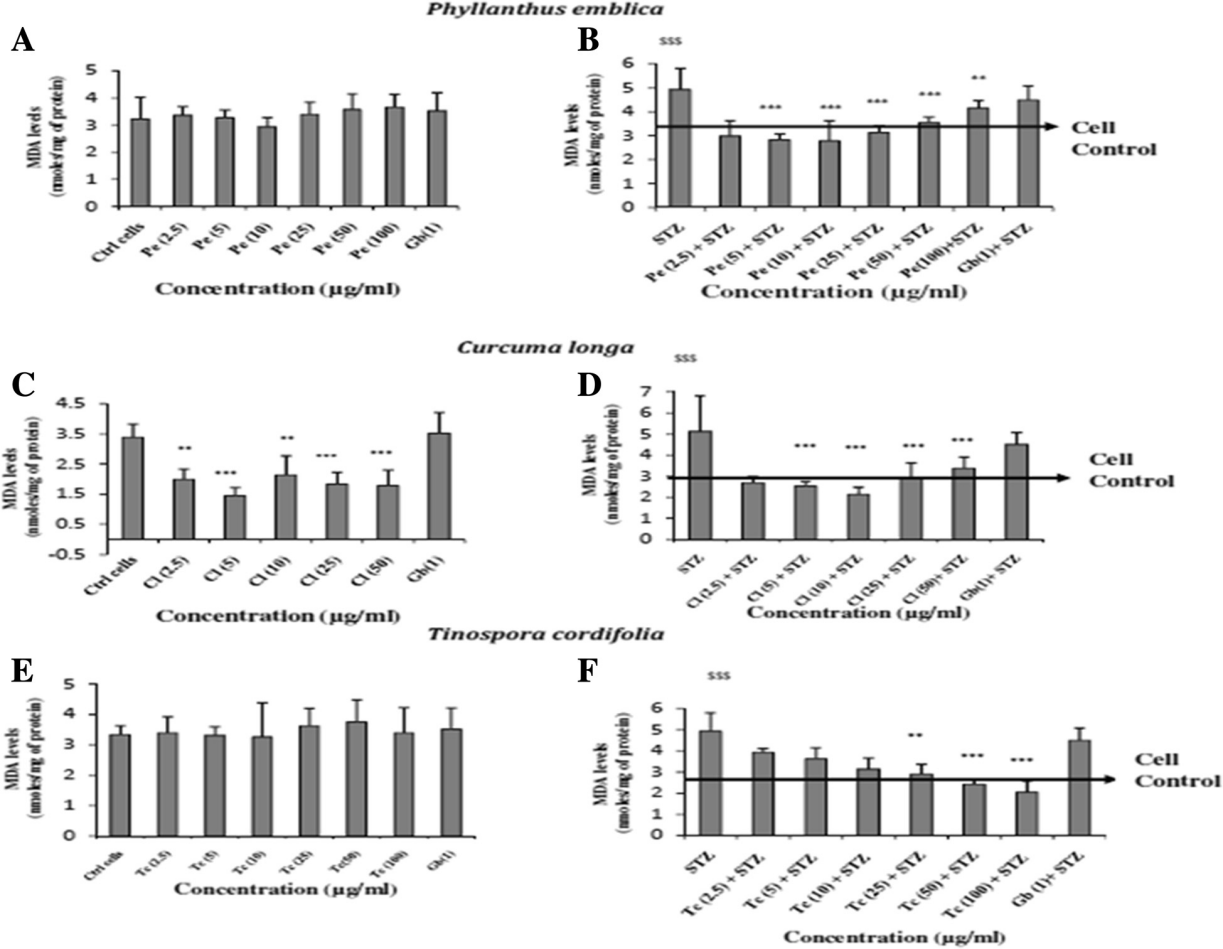
Effect of selected medicinal plants on MDA release from RINm5F cells in the absence (A,B,C) and presence (D,E,F) of STZ.

The first variable estimated was effect on lipid peroxidation in terms of MDA release to assess the anti-oxidant potential against the oxidative stress generated by STZ. The effect of plants *per se* on MDA release to assess the anti-oxidant potential of the plants was initially studied. As seen in Figure 1A, *Phyllanthus emblica* showed a dose dependent increase in MDA levels as compared to the control RIN cells. *Curcuma longa* showed a significant dose dependent decrease in MDA levels as compared to control cells demonstrating its anti-oxidant potential (Figure 1C). These results are in agreement with reported literature that *Curcuma longa* exhibits anti-oxidant activity [20].*Tinospora cordifolia per se* showed no effect on the MDA release (Figure 1E).

As expected, incubation of RIN cells with 2 mM STZ caused a significant increase in MDA levels as compared to untreated cells demonstrating oxidative stress. However in presence of STZ, *Phyllanthus emblica* showed a significant decrease in MDA release at lower concentrations *i.e.,* 2.5 to 10 μg/ml showing protection to the RIN cells against the stress induced *per se* by STZ (Figure 1B). This effect was contrary to the effect exhibited by *Phyllanthus emblica.* This is probably reflective of the *rasayana* property of *Phyllanthus emblica*. The property of enhancing physiological conditions and restoring balance or equilibrium in pathological conditions is called a *rasayana*[21]. *Phyllanthus emblica* is one such plant which is said to be a *rasayana* plant [22]. However the amount of protection exhibited at higher concentrations was less as compared to that seen with the lower concentrations.

*Curcuma longa* also demonstrated similar results as compared to *Phyllanthus emblica* showing maximum protection to RIN cells at 10 μg/ml against the stress induced by STZ (Figure 1D). *Tinospora cordifolia* showed a dose dependent decrease in MDA release in STZ treated RIN cells at all the concentrations tested with the maximum decrease seen at the highest concentration tested (Figure 1F). Although *Tinospora cordifolia too* is a *rasayana* plant, unlike *Phyllantus emblica,* it showed no effect *per se* on MDA release in untreated RIN cells. It however protected against the oxidative stress induced by STZ by demonstrating a decrease in MDA levels. This difference in activity exhibited by the two *rasayana* plants may be due to the type of extract used in the study. The increase in MDA levels at higher doses of *Phyllantus emblica per se* or in presence of STZ may be due to the presence of polar substances in the hydro-alcoholic extracts of *Phyllantus emblica*, which interfered with the anti-oxidant effect thus affecting the degree of protection. The results of our study also highlight the fact that *Ayurveda* recommends the use of decoction of plant extracts prepared in water for better activity. Glibenclamide used as the standard in the assay showed an increase in MDA release *per se* and also in the presence of STZ. Similar increase in MDA levels has also been reported in a previous study wherein MDA levels from the tissues of diabetic rats treated with Glibenclamide was estimated [23]. Although there are reports of the antioxidant effects of sulfonylureas in diabetic subjects, the effect is more prominent with glipizide than with Glibenclamide [24-26].

### Effect on apoptosis

**Figure 2:**
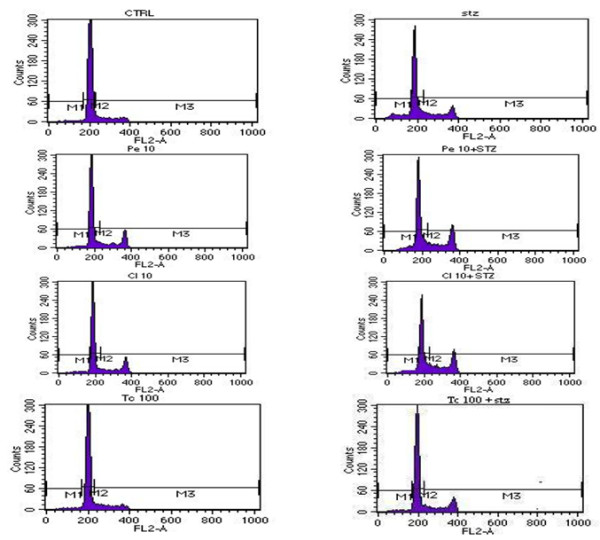
**DNA Histograms of RINm5F cells treated with selected plants in presence and absence of STZ. ** RINm5F cells were treated with different concentrations of plants extracts in presence and absence of STZ. Apoptosis was measured using cell cycle analysis from the DNA histograms generated on the Flow Cytometer followed by PI staining. All values are indicative of Mean ± SD. **p < 0.01;***p < 0.001 as compared to Untreated cells, ^$^p < 0.05; ^$$^p < 0.01; as compared to STZ untreated cells. (ANOVA followed by post hoc test).

The second variable studied was the potential of these plants to modulate apoptosis. It is reported that DNA fragmentation is a characteristic pattern of apoptotic cell death in various cell types, including β-cell lines [27-29]. RIN cells treated with the plant extracts in presence/absence of STZ were subjected to PI staining and run on a Flow cytometer to detect apoptosis using Cell Cycle analysis. As seen in Table 1 and Figure 2, STZ induced apoptosis as observed as a significant increase in the sub G0 (apoptotic) population marked as M1 (25.48 ± 3.77) as compared to untreated RIN cells (8.18 ± 1.41) in the histogram with a subsequent decrease in G0/G1 population marked as M2 (39.13 ± 6.69) as against the untreated RIN cells (52.46 ± 6.99).

**Table 1:** Effect of selected medicinal plants on apoptosis induced by STZ in RINm5F cells

**Groups under study**	**% Cells in Pre G1 (M1)**	**% Cells in G0, G1 (M2)**	**% Cells in G2,M,S (M3)**
Cell control	8.18 ± 1.41	52.46 ±6.99	26.419 ±2.56
Cells + *Pe* (2.5 μg/ml)	11.81 ±2.98	52.03 ±17.41	27.51 ± 9.19
Cells + *Pe* (5 μg/ml)	11.51 ±2.98	51.25 ±3.22	27.75 ±3.53
Cells + *Pe* (10 μg/ml)	16.03 ±4.52^*^	48.01 ±8.50	25.60 ±4.66
Cells + *Pe* (25 μg/ml)	17.39 ±1.82	49.05 ±5.53	25.97 ±1.96
Cells + *Pe* (50 μg/ml)	18.33 ±1.62^*^	45.79 ±3.17	28.47 ±1.99
Cells + *Pe* (100 μg/ml)	20.63 ±1.60^***^	44.01 ±2.35	26.29 ±1.58
Cells + STZ	25.48 ± 3.77^***^	39.13 ±6.69^*^	36.18 ±8.48
Cells + *Pe* (2.5 μg/ml) + STZ	19.25 ±3.45	41.25 ±6.69	31.02 ±8.75
Cells + *Pe* (5 μg/ml) + STZ	15.29 ±4.05^$$$^	37.86 ±6.77	34.75 ±8.34
Cells + *Pe* (10 μg/ml) + STZ	11.77 ±2.31^$$$^	45.28 ±5.61	29.13 ±7.98
Cells + *Pe* (25 μg/ml) + STZ	16.04 ±1.42^$$$^	38.65 ±3.43	35.40 ±2.99
Cells + *Pe* (50 μg/ml) + STZ	18.34 ±1.23^$^	36.89 ±4.33	29.86 ±5.26
Cells + *Pe* (100 μg/ml) + STZ	23.35 ±1.80	36.90 ±4.15	30.29 ±4.29
Cell control	9.58 ± 2.48	56.03 ± 2.65	25.51 ± 2.05
Cells + *Cl* (2.5 μg/ml)	11.05 ± 2.89	51.28 ± 3.32	26.82 ± 4.27
Cells + *Cl* (5 μg/ml)	10.36 ± 3.94	51.24 ± 2.88	28.41 ± 3.41
Cells + *Cl* (10 μg/ml)	10.94 ± 3.78	51.75 ± 5.09	28.81 ± 4.81
Cells + *Cl* (25 μg/ml)	12.98 ± 2.72	46.02 ± 7.77	30.61 ± 1.71
Cells + *Cl* (50 μg/ml)	14.7 ± 2.06	48.61 ± 4.63	31.94 ± 2.61
Cells + STZ	23.37 ± 4.51^***^	41.38 ± 7.44	32.21 ± 6.86
Cells + *Cl* (2.5 μg/ml) + STZ	17.14 ± 4.75^$$^	41.94 ± 5.02	31.21 ± 8.91
Cells + *Cl* (5 μg/ml) + STZ	15.77 ± 5.13^$$$^	40.82 ± 2.87	32.73 ± 7.59
Cells + *Cl* (10 μg/ml) + STZ	12.74 ± 3.07^$$^	44.60 ± 3.09	29.62 ± 7.73
Cells + *Cl*(25 μg/ml) + STZ	18.74 ± 2.44	42.42 ± 1.93	35.47 ± 1.38
Cells + *Cl* (50 μg/ml) + STZ	19.13 ± 3.99	40.18 ± 3.48	32.40 ± 1.67
Cell control	5.87 ± 1.70	53.22 ± 11.81	26.98 ± 7.17
Cells + *Tc* 2.5 μg/ml	7 ± 1.57	57.79 ± 13.81	23.90 ± 6.57
Cells + *Tc* 5 μg/ml	5.92 ± 1.91	59.32 ± 13.48	23.98 ± 7.96
Cells + *Tc* 10 μg/ml	7.12 ± 2.22	57.12 ± 12.35	24.58 ± 7.44
Cells + *Tc* 25 μg/ml	7.62 ± 2.17	57.13 ± 3.45	25.73 ± 6.11
Cells + *Tc* 50 μg/ml	7.07 ± 2.44	60.44 ± 3.89	20.03 ± 5.79
Cells + *Tc*100 μg/ml	10.16 ± 5.27	56.95 ± 2.47	28.68 ± 0.69
Cells + STZ	24.69 ± 2.19^***^	40.01 ± 9.17^*^	26.28 ± 1.67
Cells + *Tc*2.5 μg/ml + STZ	26.69 ± 1.34	51.35 ± 6.22	18.73 ± 8.09
Cells + *Tc* 5 μg/ml + STZ	25.86 ± 1.83	35.08 ± 10.81	34.82 ± 6.61
Cells + *Tc* 10 μg/ml + STZ	21.65 ± 3.34	43.86 ± 9.22	25.21 ± 8.91
Cells + *Tc* 25 μg/ml + STZ	16.77 ± 3.65^$$^	43.48 ± 2.13	31.36 ± 3.86
Cells + *Tc* 50 μg/ml + STZ	14.16 ± 1.69^$^	42.86 ± 6.94	28.54 ± 6.12
Cells + *Tc*100 μg/ml + STZ	14.05 ± 1.80^$$^	48.64 ± 1.3	36.12 ± 1.78
Cell control	11.87 ± 3.70	53.22 ± 11.81	26.98 ± 7.17
Cells + *Gb (1* μg/ml)	12.6 ± 2.39	49.79 ± 9.11	26.87 ± 6.33
Cells + STZ	28.5 ± 8.85^***^	40.24 ± 6.71^*^	28.42 ± 8.30
Cells + STZ + *Gb (1* μg/ml)	19.91 ± 5.05^$^	47.3 ± 5.92	25.84 ± 3.12

RINm5F cells were treated with different concentrations of plants extracts in presence and absence of STZ. Apoptosis was measured using cell cycle analysis from the DNA histograms generated on the Flow Cytometer followed by PI staining. All values are indicative of Mean ± SD. *p < 0.05;***p < 0.001 as compared to Untreated cells, ^$^p < 0.05; ^$$^p < 0.01; ^$$$^p < 0.001 as compared to STZ treated cells. (ANOVA followed by post hoc test).

In the absence of STZ, *Phyllanthus emblica* increased the number of cells in the sub G0 (apoptotic) cell population as compared to untreated control, indicating beta cell damage. However, when administered in the presence of STZ, *Phyllanthus emblica* protected the RIN cells against STZ induced damage (25.48 ± 3.77), with a dose dependent effect with maximum and significant protection at 10 μg/ml (11.77 ±2.31) subsequently increasing number of cells in G0/G1 population marked as M2 (45.28 ±5.61) as against the STZ treated RIN cells (39.13 ±6.69). Higher concentrations showed protection but to a lesser extent. The results once again confirmed the *rasayana* property of *Phyllanthus emblica*.

As reported in Table 1, incubation of RIN cells with *Curcuma longa (Cl)* alone *per se* showed no effect in the sub G0 (apoptotic) population as compared to the control RIN cells. However, when combined with STZ treatment, it showed a decrease in the sub G0 (apoptotic) cell population with maximum effect at 10 μg/ml (10.94 ± 3.78) as compared to the cells treated with STZ alone (23.37 ± 4.51). Thus, it appeared to protect the RIN cells against the damage induced by STZ by increasing the number of cells in G0/G1 population marked as M2 (51.75 ± 5.09) as against the STZ treated RIN cells (44.60 ± 3.09).

Treatment of RINm5F cells with *Tinospora cordifolia* alone did not affect the number of cells in the sub G0 (apoptotic) population except at 100 μg/ml wherein an increase in sub G0 (apoptotic) cell population was observed. *Tinospora cordifolia* also demonstrated a significant dose dependent decrease in the sub G0 (apoptotic) population with maximum effect at 100 μg/ml (14.05 ± 1.80) when administrated in the presence of STZ indicating protection against the damage induced by STZ (24.69 ± 2.19) subsequently increasing the cells in G0/G1 population marked as M2 (48.64 ± 1.3) as against the STZ treated RIN cells (40.01 ± 9.17). Results shown in Table 1.

The effect of all plants were comparable to Glibenclamide which also exhibited protection against the apoptotic damage induced by STZ by decreasing the sub G0 (apoptotic) cell population (19.91 ± 5.05 *vs*. 28.5 ± 8.85) and subsequently increasing the number of cells in G0/G1 population marked as M2 (53.22 ±11.81) as against the STZ treated RIN cells (40.24 ±6.71). These results are contrary to that seen in previous studies wherein Glibenclamide has been shown to induce apoptosis in pancreatic beta-cells or beta-cell lines under certain conditions [30]. Our explanation for the results obtained in our study is that this may be due to the difference in dose used and the time of exposure which could have influenced the action of Glibenclamide which needs to be explored further.

### Effect on insulin secretion

**Figure 3:**
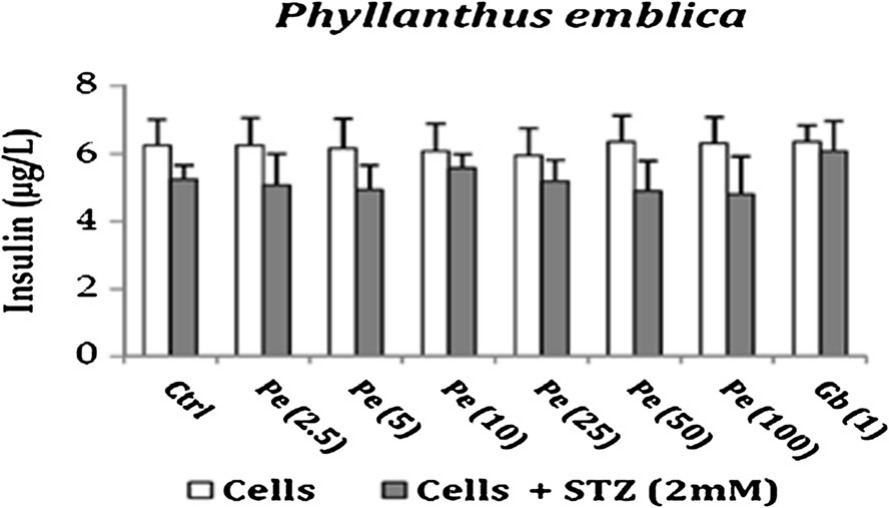
Effect of Phyllanthus emblica on Insulin release from RINm5F cells in presence and absence of STZ.

**Figure 4:**
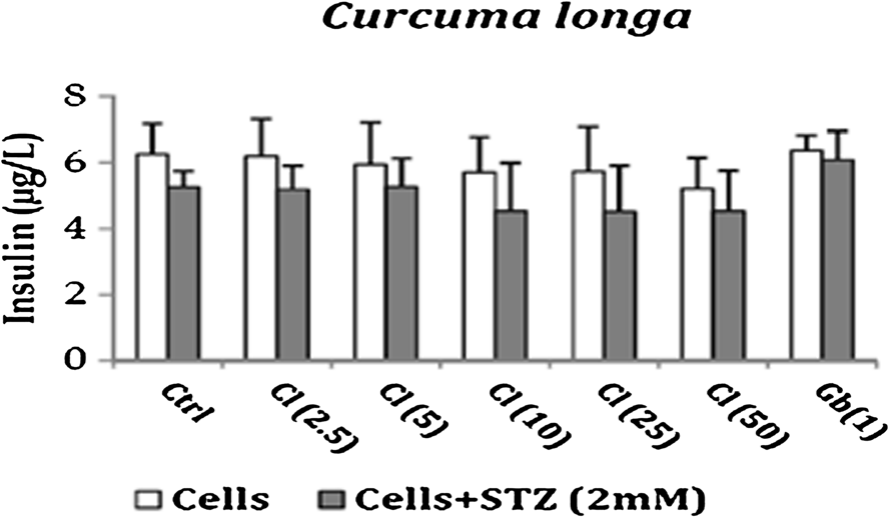
Effect of Curcuma longa on Insulin release from RINm5F cells in presence and absence of STZ.

**Figure 5:**
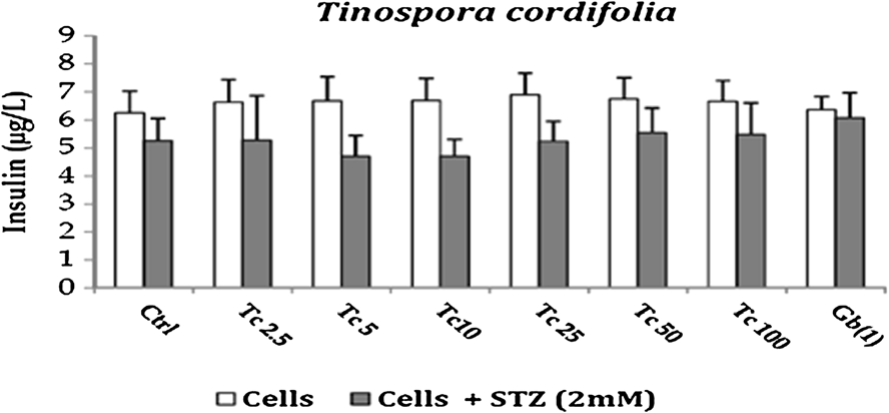
Effect of Tinospora cordifolia on Insulin release from RINm5F cells in presence and absence of STZ.

The effect of drugs on insulin secretion was evaluated as damaged pancreatic beta cells lose their capacity to secrete insulin. *Phyllanthus emblica per se* showed no effect on insulin secretion as compared to the untreated cells. However in presence of STZ, it showed an increase in insulin secretion at 10 μg/ml. This concentration is the same at which maximum anti-oxidant and anti-apoptotic effect of *Phyllanthus emblica* was observed. *Curcuma longa (Cl) per se* and in presence of STZ demonstrated a decrease in insulin secretion as compared to the untreated cells. These results emphasize the fact that *Curcuma longa* may have an effect on activation of Poly ADP ribosylation which play a role in inhibiting insulin secretion [31]. *Tinospora cordifolia (Tc) per se* increased insulin release as compared to the untreated cells. In presence of STZ, an increase in insulin release was observed at higher concentration *i.e.,* 100 μg/ml however the effect was not significant. Glibenclamide *per se* showed an increase in insulin secretion and also increased the insulin release from STZ treated cells thus confirming its mode of action which is mainly by stimulating the cells in the pancreas that produce insulin. The results are represented in Figures 3, 4 and 5.

The results of MDA release demonstrate the protective effect of the plants against the oxidative stress induced by STZ once again confirming the reported anti-oxidant potential of the plants thereby strengthening the evidence that these plants can be used to reduce the oxidative stress generated in diabetes and further help in delaying the development of diabetic complications.

Hydro-alcoholic extract of *Phyllanthus emblica* consists of presence of phyto-constituents *viz.,* alkaloids, glycosides, reducing sugars, tannins, resins, saponins, sterols and fixed oils. Vitamin C, the major phyto-constituent of *Phyllanthus emblica* is a known anti-oxidant [32]. Hence we can say that Vitamin C may contribute to the anti-oxidant activity noted in terms of lipid peroxidation exhibited by *Phyllanthus emblica*. Gallic acid and Ellagic acid, which are well-known natural antioxidants forms the major phyto-constituents of *Phyllanthus emblica*. Gallic acid has been reported to possess anti-apoptotic and insulin secretagogue activity in RINmF cells [33]. Ellagic acid is also reported to exhibit both antioxidant activity in V79-4 cells and apoptosis-inducing activity in HOS cells [34]. Thus the anti-oxidant and anti-apoptotic activity possessed by *Phyllanthus emblica* may be due to the presence *of Gallic acid and Ellagic acid*. Curcumin, the major active constituent present in hydro-alcoholic extract of *Curcuma longa,* exhibits anti-oxidant as well as anti-apoptotic activity [35,36]. Thus, we can conclude that the anti-apopototic and anti-oxidant effect shown by *Curcuma longa* may be attributed to the presence of Curcumin. Alkaloids, tannins, cardiac glycosides, flavonoids, saponins, and steroids as the major phyto-constituents [37] of *Tinospora cordifolia* have been reported to play an anti-diabetic role. Alkaloids like *Berberine* are reported to have anti-oxidant and anti-apoptotic activity [38,39]. Glycosides like *Tinocordiside* and *Tinocordifolioside h*ave been reported to have anti-oxidant and hydroxyl radical scavenging activities in Swiss albino mice [40].Hence the anti-oxidant and anti-apoptotic activity exhibited by *Tinospora cordifolia* in our study may be due to the presence of these constituents.

The data on cell cycle analysis clearly revealed the role of STZ as an apoptotic agent, and the effect seen with the plant extracts revealed the role of plants as anti-apoptotic agents suggesting that the observed growth-inhibitory effect of STZ in RIN cells could be ameliorated by addition of these plants. Also the results of cell cycle analysis of all the plants were similar to the results of MDA release demonstrating that the anti-oxidant potential exhibited by the plant extracts facilitated the protection against the oxidative stress induced by STZ.

From the above results the probable mechanism of action of *Curcuma longa* and *Tinospora cordifolia* may be through their anti-oxidant potential which was confirmed in our study through their ability to decrease lipid peroxidation in the presence of STZ thus showing protection against the induced oxidative damage. Although *Phyllanthus emblica per se* showed an increase in lipid peroxidation it protected the RIN cells against the damage induced by STZ proving its *rasayana* property. The second mode of anti-diabetic action may be through its anti-apoptotic effect as seen by the protection offered by *Phyllanthus emblica, Curcuma longa* and *Tinospora cordifolia* in abrogating apoptosis induced by STZ resulting in a cytogram with similar profile to control cells. Their anti-apoptotic action can help in preservation of the residual β-cell mass with minimal effect seen on insulin secretion. The importance of the progressive loss of pancreatic β-cell reduction in the course of type 2 Diabetes has been the focus of therapeutic targets in the development of novel and potential drugs acting by enhancing pancreatic β-cell growth and/or survival. Thus prolonged administration of these plants may expand pancreatic β-cell mass, leading to increased insulin secretion and improved glycemic control. These plants may thus have a beneficial effect in diabetic individuals through their anti-oxidant and anti-apoptotic actions.

Although the study reveals the mechanism of action of the selected plants it is essential to further elucidate the underlying molecular mechanism for the anti-diabetic action using *in vivo* models of diabetes and its associated complications. Also isolation and identification of active constituents from these plants, preparation and standardization of the dose, pharmacological and toxicological evaluation of the active principle will help in developing these plants into potential anti-diabetic drugs which after clinical assessment can be incorporated in the anti diabetic drug armamentarium.

## Conclusion

The present study confirms the protective effect of the selected medicinal plants against the oxidative damage induced by STZ. This is the first study to reveal the anti-diabetic mechanistic actions of *Phyllanthus emblica, Curcuma longa* and *Tinospora cordifolia* which is exerted through their anti-oxidant and anti-apoptotic potential.

## Competing interests

The authors declare that they have no competing interests.

## Authors’ contributions

SAK, designed and performed all the experiments, interpreted the results and drafted the manuscript. RPM supervised the experimental work, reviewed and interpreted the results, revised the manuscript. UMT was responsible for the concept, overall coordination of the study and review of the manuscript for intellectual inputs. All authors read and approved the final manuscript.

## Pre-publication history

The pre-publication history for this paper can be accessed here:

http://www.biomedcentral.com/1472-6882/13/257/prepub
